# Supplementary material for: The Blockade of Tumoral IL1β-Mediated Signaling in Normal Colonic Fibroblasts Sensitizes Tumor Cells to Chemotherapy and Prevents Inflammatory CAF Activation
Source: Int J Mol Sci. 2021 May 7;22(9):4960. doi: 10.3390/ijms22094960 (PMC8125420; doi:10.3390/ijms22094960)

Supplementary data

The blockade of tumoral IL1β-mediated signaling in normal colonic fibroblasts sensitizes tumor cells to chemotherapy and prevents inflammatory CAF activation

Natalia Guillén Díaz-Maroto[1,3], Gemma Garcia-Vicien[1,3], Giovanna Polcaro[2], María Bañuls[1], Nerea Albert[1,3], Alberto Villanueva[1,3], David G. Molleví[1,3]*.

1. ProCURE, Program Against Cancer Therapeutic Resistance, Catalan Institute of Oncology, IDIBELL, L'Hospitalet de Llobregat, Catalonia, Spain.

2. Universita degli Studi del Sannio, Dipartimento Scienze e Tecnologie, Benevento, Italy

3. ONCOBELL Program, IDIBELL, L'Hospitalet de Llobregat, Catalonia, Spain.

Supplementary results

Soluble factors secreted by normal colonic fibroblasts upon IL1β stimulation

We explored IL1β targets in a cytokine array that provides information about 174 cytokines or growth factors. Of these 174 soluble factors, only 42 were upregulated with IL1β, and their levels were controlled with a blocking antibody (direct targets of IL1β). These molecules are depicted in Supplementary Figure 2. Those with the highest concentrations after IL1β were IL6, IL8, CCL2, CXCL1, CCL7, CCL11 and TNFRSF11B, most of which had been previously reported to induce chemoprotection to various cytotoxic drugs. Nevertheless, we cannot discount the relevance of other cytokines expressed at lower concentrations but with greater overexpression compared with controls such as CCL8, CCL5, CXCL5 and IL12. We believe that the observed protective effect cannot be attributed to a single factor and that the phenotype induced is probably the consequence of the action of several soluble factors. In addition, 12 cytokines displayed a monotonic increase, from controls to IL1β-stimulated and IL1β-stimulated plus neutralising antibody. These were: VEGFR3 ERBB3, IGF2, CD170, CD80, MMP1, MMP9, IL1R2, IL13RA2, IL2RB, CXCL12 and MCSF-R. Nonetheless, the trigger for the secretion of IL1β by tumor cells has not been fully elucidated. Regarding the cytokines

secreted by NCFs at basal levels (black bars in Supplementary Figure 2), we highlight CCL2 and CCL11 as possible candidates since they were secreted at higher concentrations. However, when we stimulated DLD1 cells with CCL2 or CCL11, no IL1β mRNA induction was observed.

As expected, most of the IL1β targets stimulated JAK/STAT and AKT pathways in tumors cells. This activation can be reversed by neutralizing the binding of IL1β to their receptors in NCFs (Figure 4A). In addition, CM from IL1β-treated fibroblasts induces the overexpression of Cyclin D1 and cMyc, which might help explain how IL1β-soluble targets influence the cell-cycle progression and chemoresistance observed in tumor cells when treated with L-OHP (Figure 4B).

Supplementary figure legends

Supplementary Figure S1

Cytokine array (RayBiotech) assaying the levels of 174 cytokines and growth factors in normal colonic fibroblasts, IL1β-stimulated normal colonic fibroblasts [10 ng/ml] or IL1β-stimulated normal colonic fibroblasts plus a neutralising IL1β antibody. Of the 174 soluble factors, 42 were induced by IL1β and their expression counterbalanced by the blocking antibody. Conversely, VEGFR3, IGF-2, ERBB3, Tie-1, IL1R2, CXCL12, CD170, CD80, MMP1, MMP9, and IL2RB increased with IL1β stimulation but even more with IL1β plus neutralising antibody.

Supplementary Figure S2

Lentiviral vector pGIPZ (Dharmacon) used for silencing of IL1β in tumor cells (HT29). We checked five different hairpins, being V3LHS_321411 and 321412 those showing higher percentages of inhibition.

We checked by means of RT-PCR the percentage of IL1β mRNA silencing of clon V3LHS_321411 (white bar) compared to non-silencing vector (grey bar) and wild-type cells (black bar). Expression was normalized against GAPDH. The percentage of silencing was standardized in relation to cells with the control vector (wild-type 100%, non-silencing 95.67% and HT29shIL1β 17.67%%).

Supplementary figure S3

RAW data corresponding to all the WB membranes used in the manuscript.

Supplementary figure 1

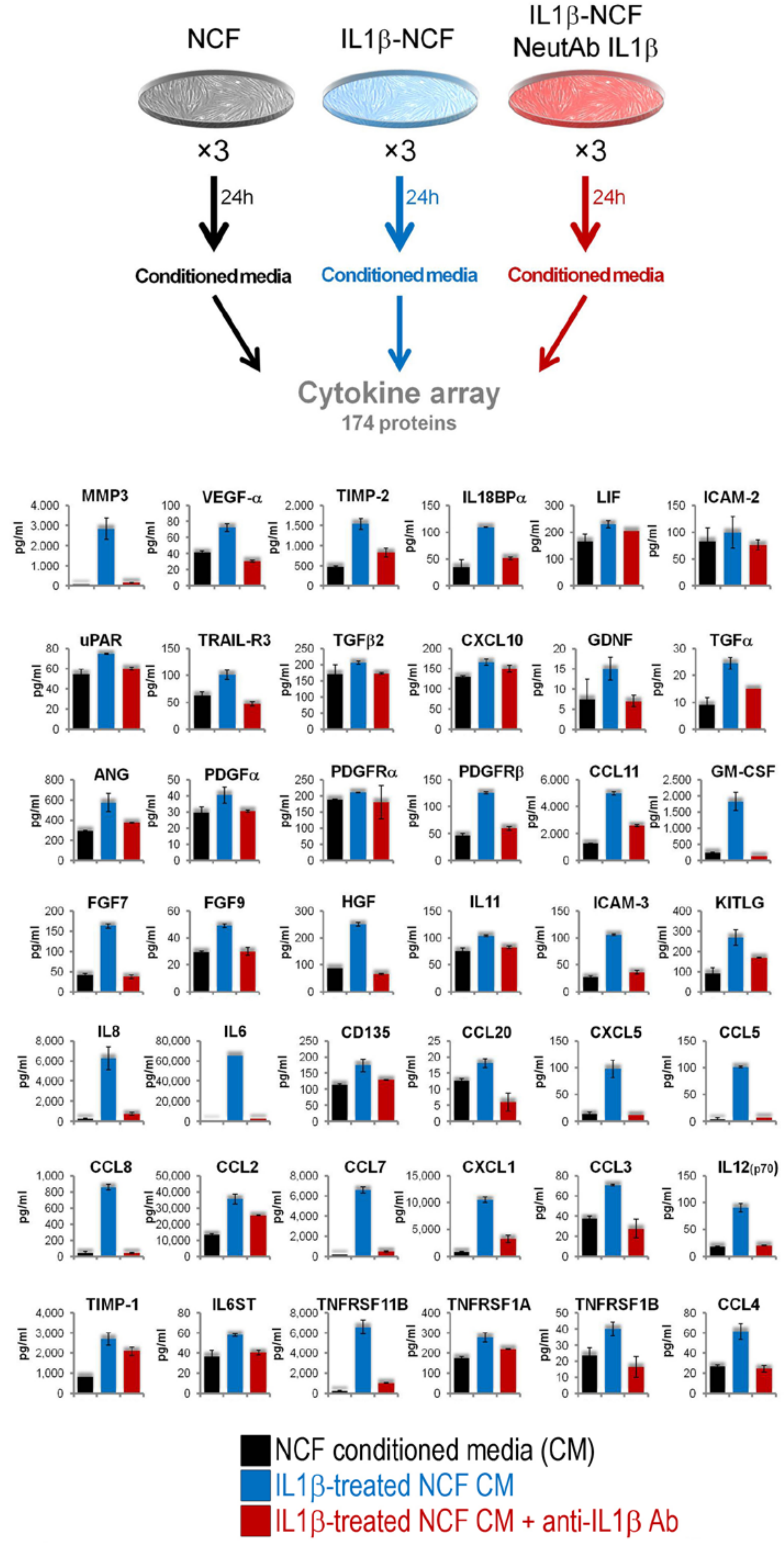

Supplementary figure 2

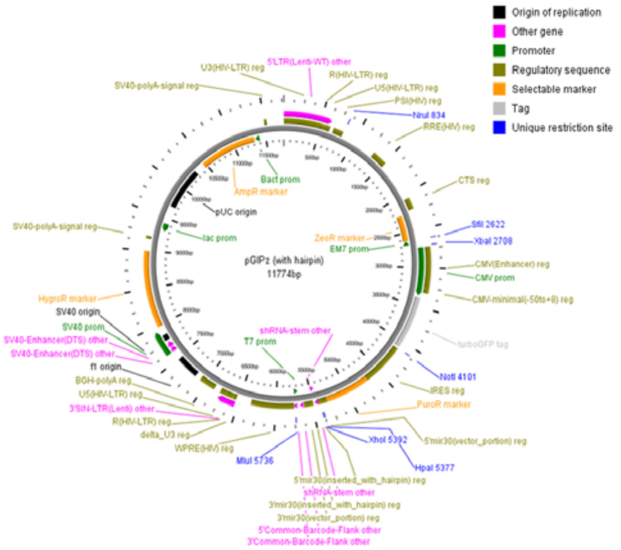

V3LHS_321411 mature antisense TTGGGATCTACACTCTCCA
V3LHS_321412 mature antisense TGTTGAAGACAAATCGCTT

Sequencing primer: 5' - GCATTAAAGCAGCGTATC - 3'

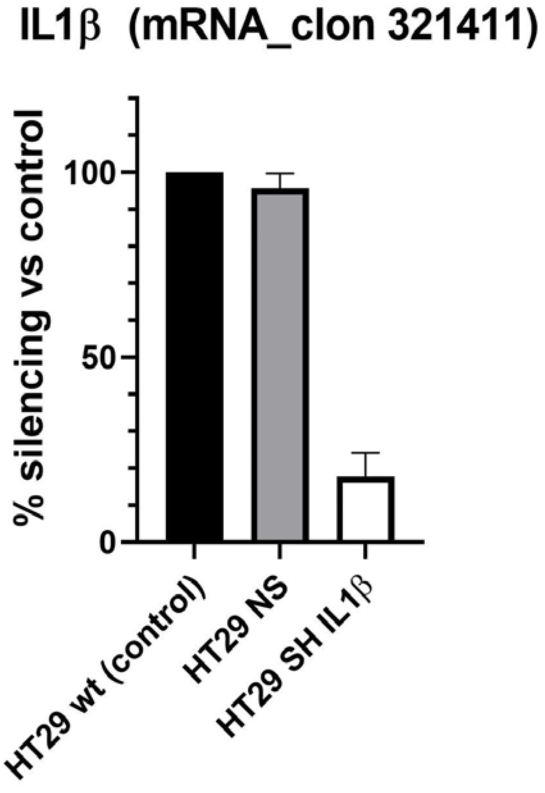

Supplementary figure 3

membranes corresponding to Figure 2A

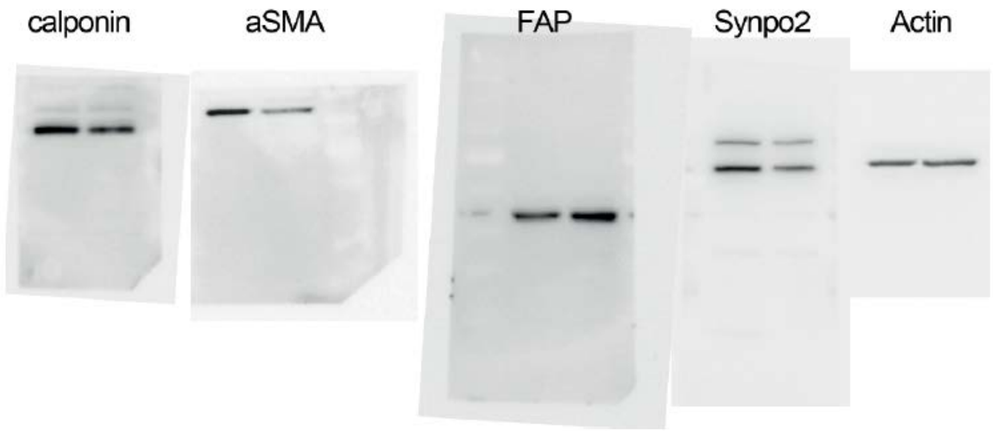

membranes corresponding to Figure 2J

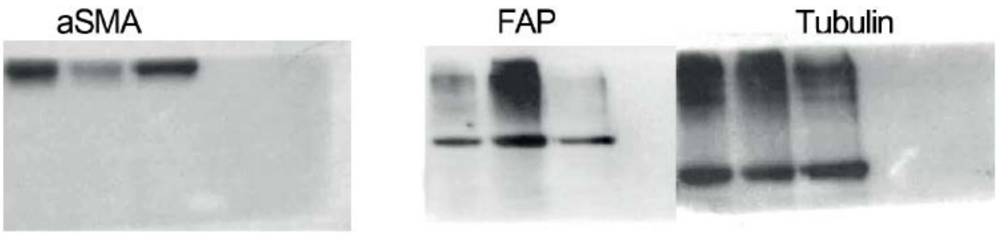

membranes corresponding to Figure 4D

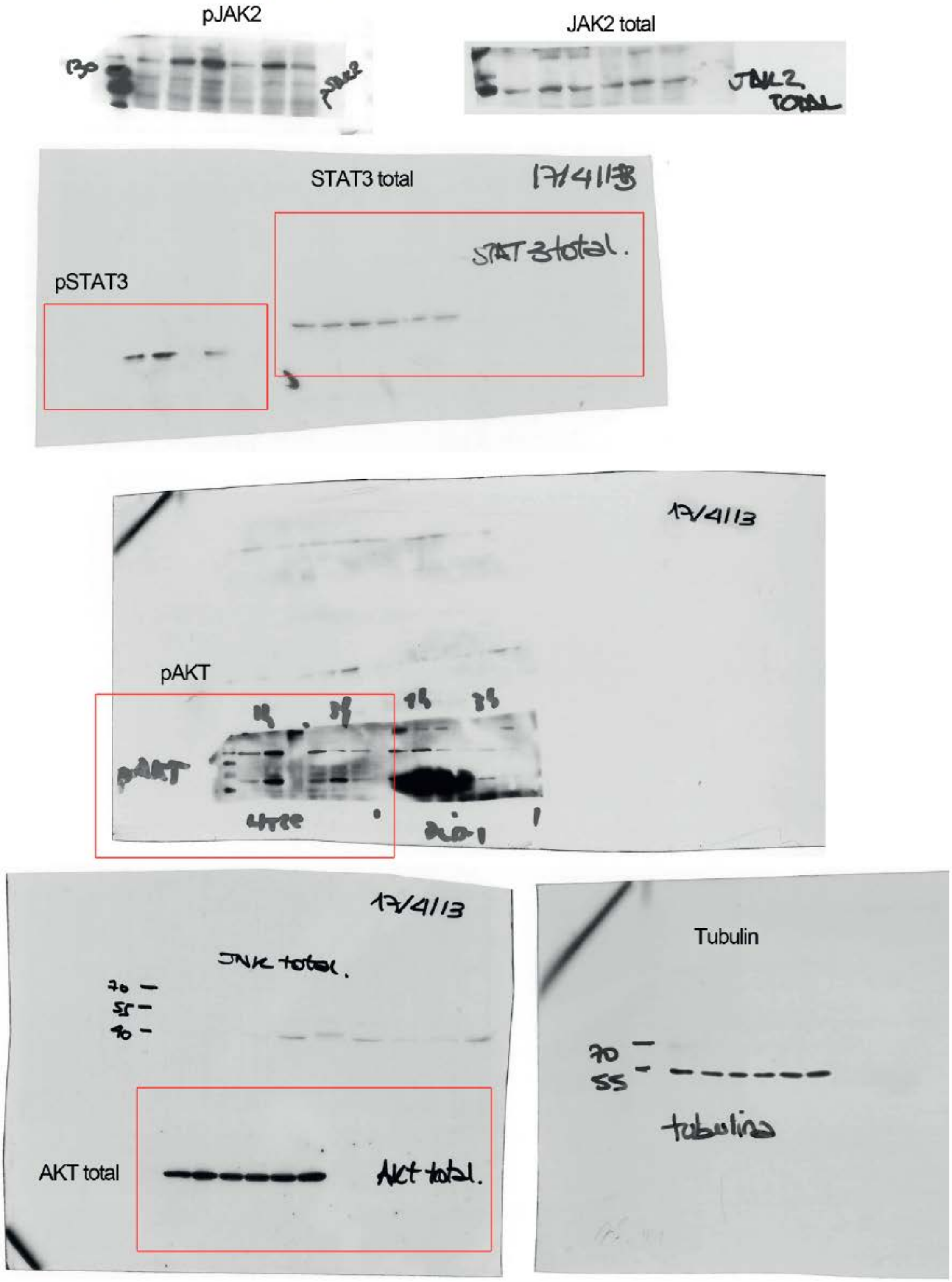

membranes corresponding to Figure 4E

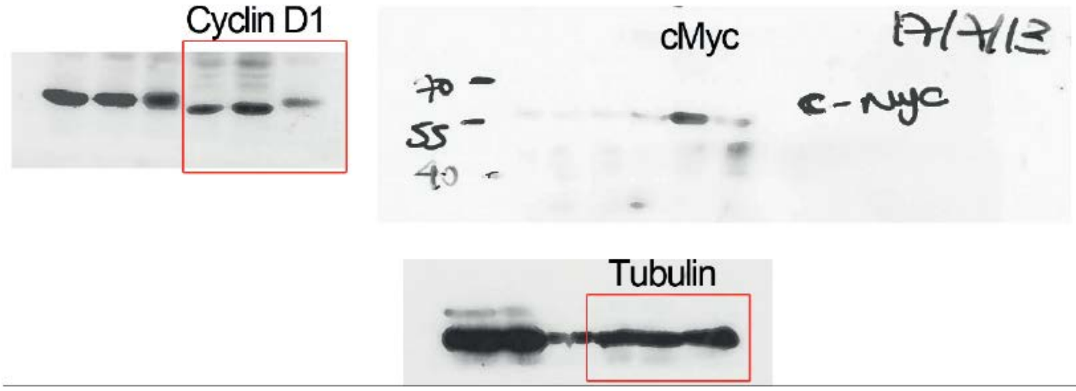

Supplement: Supplementary file 1 [file ijms-22-04960-s001.zip › ijms-1193062-supplementary.pdf]
